# Six-month outcomes in lung cancer patients surviving ICU admission: results from a multinational multicenter study

**DOI:** 10.1186/cc13244

**Published:** 2014-03-17

**Authors:** M Soares, JF Timsit, G Burghi, C Irrazabal, N Pattison, E Tobar, BF Almeida, UV Silva, LC Azevedo, JI Salluh, E Azoulay

**Affiliations:** 1 DOr Institute for Research and Education - DOR, Rio de Janeiro, Brazil; 2 Hôpital A. Michallon Chu de Grenoble, France;Hopital A. Michallon Chu de Grenoble, France;Hopital A. Michallon Chu de Grenoble, France;Hopital A. Michallon Chu de Grenoble, France; 3 Hospital Maciel, Montevideo, Uruguay; 4 Instituto Alexander Fleming, Buenos Aires, Argentina; 5 Royal Brompton NHS Foundation Trust, London, UK; 6 Hospital Clinico Universidad de Chile, Santiago, Chile; 7 Hospital A.C. Camargo, São Paulo, Brazil; 8 Fundação Pio XII - Hospital do Câncer de Barretos, Barretos, Brazil; 9 Hospital Sírio Libanês, São Paulo, Brazil; 10 Hôpital Saint Louis, Paris, France

## Introduction

Information about lung cancer patients surviving critical illnesses is very scarce. Our aim was to evaluate the outcomes and continuing of anticancer treatments in lung cancer patients surviving ICU admission.

## Methods

Secondary analysis of a prospective multicenter study including patients admitted for >24 hours to 22 ICUs in six countries from Europe and South America during 2011. Readmissions and patients in cancer remission >5 years were excluded. Logistic regression was used to identify predictors for hospital mortality.

## Results

A total of 449 patients (small-cell (SCLC) = 55; non-SCLC = 394)) were admitted to ICUs, and out of them 275 (SCLC = 29; NSCLC = 246) were discharged alive from the hospital. Among them, 200 (73%) patients were alive and 72 (26%) had died at 6 months; three (1%) patients were lost to follow-up. Mortality rates were far lower in the patient subset with nonrecurrent/progressive cancer and a good performance status (PS), even those with sepsis, multiple organ dysfunctions, and need for ventilatory support. Cancer recurrence or progression occurred in 53 (26%) hospital survivors. Anticancer treatments were recommended for 108 (39%) hospital survivors and administered to 102. Treatments used were variable combinations of surgical resection (7%), radiation therapy (34%), and chemotherapy (80%). The initial treatment plan required reduction or modification in 35 (34%) patients. Post-hospital mortality was nonsignificantly lower in the patients given the initial treatment plan than in the other patients (17% vs. 32%, *P = *0.065). Poor PS was the only factor associated with a lower probability of receiving the initial treatment plan (OR = 0.20; 95% CI, 0.05 to 0.87; *P = *0.032). At 6 months, 71% patients were at home, 15% were hospitalized, and 7% were in hospice care; the location was unknown for 6% patients. PS at 6 months was 3 to 4 in 19 (9.5%) survivors.

## Conclusion

Post-hospital mortality in critically ill lung cancer patients is relatively high and many patients require anticancer treatments after discharge. PS before ICU admission is a major determinant of both mortality and ability to receive optimal anticancer treatment in these patients.

